# Comorbidities and Pregnancy-Related Risk Factors in Patients with Severe Maternal Morbidity: Application of a Validated Obstetrical Comorbidity Scoring System to a Surveillance-Identified Population

**DOI:** 10.3390/healthcare13182351

**Published:** 2025-09-18

**Authors:** Carrie Wolfson, Jessica Tsipe Angelson, Alexandra D. Forrest, Erin D. Michos, Saifuddin Ahmed, Abimbola Aina-Mumuney, Andreea A. Creanga

**Affiliations:** 1 Department of International Health, Johns Hopkins Bloomberg School of Public Health, Baltimore, MD 21205, USA; acreang3@jhu.edu; 2 Department of Population, Family and Reproductive Health, Johns Hopkins Bloomberg School of Public Health, Baltimore, MD 21205, USA; jangels1@jhu.edu (J.T.A.); sahmed3@jhu.edu (S.A.); 3 Department of Gynecology and Obstetrics, Johns Hopkins School of Medicine, Baltimore, MD 21205, USA; aforres9@jh.edu (A.D.F.); aaina1@jhmi.edu (A.A.-M.); 4 Department of Medicine (Cardiology), Johns Hopkins School of Medicine, Baltimore, MD 21205, USA; edonnell@jhmi.edu

**Keywords:** severe maternal morbidity, comorbidity, risk adjustment

## Abstract

**Background/Objectives**: Patient characteristics—especially comorbidities—influence the risk of severe maternal morbidity (SMM). Recent efforts have sought to derive an obstetric comorbidity score to be used for case-mix adjustment. We assess the use of a validated obstetric comorbidity index among patients with SMM and identify additional conditions that could be included in the index. **Methods**: We applied a validated obstetrical comorbidity scoring system to patients identified through Maryland’s SMM Surveillance and Review program, using chi-square analyses to compare prevalence of comorbidities by primary causes of SMM. We compared mean and median comorbidity score by hospital level of care and adverse outcomes (length of stay, volume of blood product transfusion, intensive care unit (ICU) admission, transfer to a higher level of care, and neonatal intensive care unit (NICU) admission). Through the review of case data, we identified additional risk factors for SMM. Using tetrachoric correlation, we examined the degree of correlation between comorbidities from the validated index and the additional risk factors in our sample. **Results**: A total of 978 SMM events were identified and reviewed between 2020 and 2024. Mean comorbidity score was highest among patients with SMM from hypertensive disorders of pregnancy, and prevalence of index comorbidities varied by primary cause of SMM. Patients that delivered at level IV hospitals had the highest mean comorbidity scores. Scores were also higher with a length of stay ≥4 days, larger volumes of blood product transfusion, and patients whose newborns were admitted to the NICU. We identified 13 additional risk factors for consideration in future indices, finding minimal correlation between the 27 indicators in the validated index and the additional 13. **Conclusions**: Accurately identifying patient risk for SMM has important applications in both clinical settings and population-level perinatal health research.

## 1. Introduction

High rates of adverse maternal health outcomes, including maternal mortality and severe maternal morbidity (SMM), remain a critical issue for pregnancy-related healthcare in the US. SMM events, which are unexpected outcomes of labor and delivery that have short- and long-term health consequences, are experienced by approximately 50–60,000 women annually [1]. Women may be at higher risk of physical and mental health complications during the postpartum period, and adverse neonatal outcomes are more likely to occur in pregnancies impacted by SMM [2,3,4]. Approximately one in three SMM events are preventable [5,6], and, increasingly, SMM is being used as an outcome measure to evaluate quality improvement initiatives in obstetrics as well as to assess hospital performance [7,8].

Patient characteristics—especially the presence of comorbid conditions—highly influence the risk of SMM [9,10]. To this end, recent efforts have been made to derive an obstetric comorbidity score that can be used for case-mix adjustment to allow for the comparison of SMM rates across clinical settings and patient populations [11,12,13]. The most recently validated obstetric comorbidity index, by Leonard et al. (2022) (hereafter referred to as the Leonard index), was derived using the SMM identification algorithm developed by the Centers for Disease Control and Prevention (CDC) and identified 27 risk factors for SMM readily available in administrative hospital records through ICD-10-CM codes (Table A1) [13]. The index was derived using targeted causal inference integrated with machine learning and assigns a higher score to risk factors based on their importance for predicting SMM during the delivery hospitalization.

Despite prior validation, there has been limited application of existing comorbidity indices to SMM surveillance databases, which may contain more detailed clinical data than administrative hospital datasets. We applied the Leonard index to a population of patients identified through facility-based SMM surveillance in Maryland. In this population, SMM is defined as follows using American College of Obstetricians and Gynecologists (ACOG)/Society for Maternal and Fetal Medicine (SMFM) criteria: patients during pregnancy and within 42 days postpartum who are admitted to the intensive care unit and/or receive a transfusion of four or more units of blood products. This measure of SMM has fewer false positives than the CDC algorithm and is recommended for use in facility-based monitoring to identify the most severe cases of SMM that warrant review for quality improvement [14,15].

The primary aim of this study was to test whether the validated obstetric comorbidity index can be practically applied to a population of patients with SMM identified through surveillance methods and to assess variation in score and conditions by features of the SMM event. Additionally, we sought to assess how the comorbidity index score differs by hospital level of care and adverse pregnancy outcomes. Finally, we sought to identify and measure the prevalence of additional comorbidities and pregnancy-related conditions among this population that are not included in the Leonard index and to determine whether they are correlated with conditions included in the existing index.

## 2. Materials and Methods

This cross-sectional study included pregnant and postpartum patients experiencing SMM in Maryland between 2020 and 2024 in one of 26 hospitals participating in SMM Surveillance and Review program during the data collection period (specific dates of entry into the surveillance program varied across hospitals). The SMM surveillance case definition was adapted from the ACOG/SMFM recommendation for hospital-based surveillance during pregnancy or within 42 days postpartum and included the following types of patients: (a) those admitted to an intensive or critical care unit (ICU/CCU) and/or (b) those with ≥4 units of blood products transfused [14]. Detailed information about Maryland’s hospital-based SMM Surveillance and Review program is available elsewhere [5,16]. Briefly, SMM events are identified by trained nurse data abstractors in each hospital using the electronic health record and are reviewed by hospital-based committees. Data collected on each event are standardized and include structured data elements, summary case narratives, and unstructured data from preventability assessments performed by multidisciplinary hospital review committees (typically consisting of obstetricians, quality improvement specialists, nursing staff, and the data abstractors), which are entered in a common electronic database. Structured data include pre-pregnancy and pregnancy-related health conditions. Review committees determine primary and contributing causes of morbidity.

For this study, we first aligned comorbidities and pregnancy-related conditions from the abstracted case data with those from the Leonard index (Table A1) and then applied the index to derive a comorbidity index score for each patient in the population. Scores could range from 0 to 200 (in patients with the presence of all index comorbidities). Using chi-square analyses, we compared the prevalence of each comorbidity by the top four primary causes of SMM in Maryland (i.e., obstetric hemorrhage, hypertensive disorders of pregnancy, infection, and cardiovascular conditions including cardiomyopathy and stroke) and timing of SMM occurrence (i.e., antepartum, intrapartum or early-postpartum *within 8 h of delivery*, and late postpartum *>8 h after delivery*). Each SMM event could have only one primary cause, which is determined by the hospital-based review committee.

In addition, using chi-square analyses and box-plots, we compared mean and median patient comorbidity scores by hospital level of care (levels I–IV) as well as the following adverse outcomes: length of stay (<4 versus ≥4 days), ICU admission among those with blood product transfusion ≥ 4 units, units of blood products transfused (divided into quartiles: ≤4, 5–6, 6–10, ≥10 units), NICU admission among those with live birth deliveries, and transfer to a higher level of care (among those first admitted to a lower level facility).

Next, we examined the degree of correlation between comorbidities from the Leonard index in our sample. We considered a correlation coefficient (r) |<0.2| low, |0.2–0.4| moderate, and >|0.8| high. Through the review of case data and expert consultation, we identified additional candidate risk factors for SMM, which were assessed in the same fashion as those for the original index. Additional conditions and risk factors were considered if they were deemed to be common among this population of patients experiencing SMM or if they were likely to greatly increase the risk of SMM given what is known from the existing literature. All analyses were conducted in Stata version 17.

## 3. Results

Between 2020 and 2024, 978 SMM events were identified and reviewed in Maryland as part of the SMM Surveillance and Review program. Obstetric hemorrhage was the leading cause of SMM (n = 522, 53.4%), followed by hypertensive disorders of pregnancy (n = 112; 11.5%), infection (n = 84; 8.6%), and cardiovascular conditions (n = 51; 5.2%) (Table 1). Mean maternal age at the time of SMM was 32.3 years (SD, 6.6). The highest percentage of patients were non-Hispanic Black (44.4%), followed by non-Hispanic White (31.5%), Hispanic (13.6%), and Asian (7.4%). Most SMM events occurred during the delivery hospitalization (79.1%); the remaining occurred within the antepartum period during a non-delivery hospitalization (11.8%) or the postpartum period during an admission after delivery (9.0%).

Across the full sample of patients with SMM, the mean obstetric comorbidity score was 18.1 (SD, 15.0), and the score varied significantly by primary cause of SMM. Patients with SMM due to hypertensive disorders of pregnancy had the highest mean comorbidity score at 26.2 (SD, 16.4), while those with SMM due to infection had the lowest score at 11.8 (SD, 9.2) (Table 2). Through chi-square analysis, we identified patterns of conditions that occurred at higher rates in patients by primary cause of SMM. Specifically, patients with SMM due to hemorrhage had a disproportionately high rate of placental complications, including placental accreta spectrum (14.6% compared to 7.9% overall, *p* < 0.001), placental abruption (11.1% compared to 7.6% overall, *p* < 0.001), and placenta previa (9.8% compared to 5.7% in patients overall, *p* < 0.001). Patients with SMM due to hemorrhage also had higher rates of prior cesarean delivery (36.6% compared to 29.6% overall, *p* < 0.001) and advanced maternal age (≥35 years) (45.6% compared to 40.3% overall, *p* < 0.001).

Patients with SMM due to hypertensive disorders of pregnancy had higher rates of preeclampsia with and without severe features, as well as chronic hypertension. They also had higher rates of placental abruption (12.5% compared to 7.6% overall, *p* < 0.001), substance use disorder (17.9% compared to 14.2% overall, *p* = 0.006), preexisting diabetes (13.4% compared to 7.5% overall, *p* = 0.002), obesity (15.2% compared to 7.2% overall, *p* = 0.003), and preterm birth (53.6% compared to 32.0% overall, *p* < 0.001).

Patients with SMM due to infection had higher rates of substance use disorder (16.7% compared to 14.2% overall, *p* = 0.007) or none of the index comorbidities (10.7% compared to 5.5% overall, *p* < 0.001). Finally, patients with SMM due to cardiovascular conditions had higher rates of pulmonary hypertension (5.9% compared to 0.4% overall, *p* < 0.001), preexisting cardiac disease (21.6% compared to 4.2% overall, *p* < 0.001), preeclampsia without severe features (11.8% compared to 9.6% overall, *p* < 0.001), substance use disorder (25.5% compared to 10.7% overall, *p* < 0.001), and chronic hypertension (25.5% compared to 14.2% overall, *p* < 0.001).

Rates of conditions also varied by timing of SMM (Table 2). Asthma and substance use disorder occurred at higher rates in antepartum SMM events compared to intrapartum/early postpartum and late postpartum periods. Placenta accreta spectrum, placenta previa, uterine fibroids, prior cesarean delivery, and advanced maternal age occurred at higher rates in intrapartum/early postpartum SMM events. Asthma and preeclampsia with non-severe features occurred at higher rates in late postpartum SMM events.

**Figure 1:**
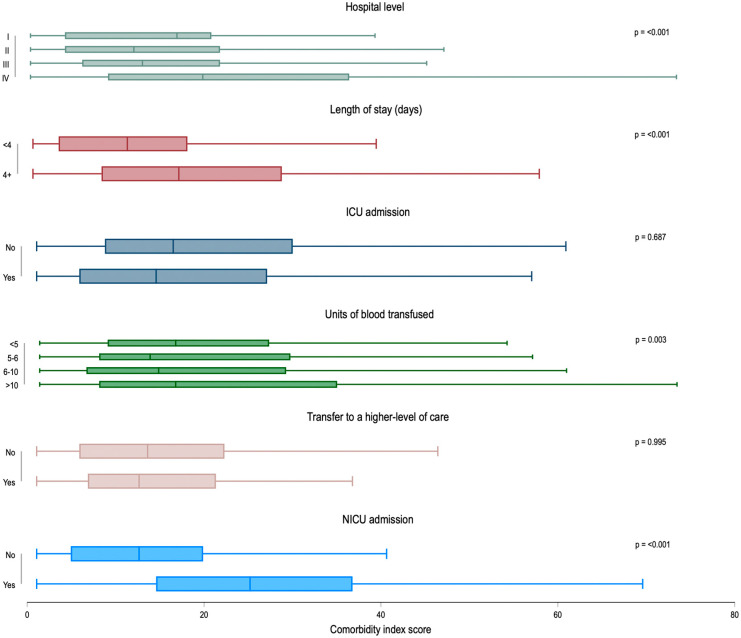
Box plot distribution of Leonard comorbidity score by hospital level of care and adverse outcomes: Maryland Severe Maternal Morbidity Surveillance and Review Program, 2020–2024 (N = 978). Note: ICU = intensive care unit; NICU = neonatal intensive care unit. ICU admission was calculated among patients who received transfusion of ≥4 units of blood products (n = 581); units of blood transfused were calculated among those with a blood transfusion of any amount (n = 612); transfer to a higher level of care was calculated among patients first admitted to a Level I–III facility (n = 657); NICU admission was calculated among patients with live birth deliveries (n = 740). Mean comorbidity score and 95% confidence intervals (CI) for Hospital Level of Care (n = 978): I = 16.6, 10.9–22.3; II = 13.7, 9.5–17.9; III = 15.4, 14.2–16.5; IV = 24.0, 22.4–25.6; length of stay (n = 978): < 4 days = 12.8, 10.9–14.6; ≥4 days = 20.0, 18.9–21.0; ICU admission (for those with blood transfusion) (n = 581): yes = 18.1, 16.0–20.1; no = 19.6, 17.9–21.4; units of blood products transfused (n = 612): 4 units or less = 17.9, 15.8–20.0; 5–6 units = 18.4, 15.8–21.1; 6–10 units = 18.9, 16.2–21.7; >10 units = 21.6, 18.9–24.2; transfer to a higher-level facility (n = 657): yes = 14.8, 11.4–18.2; no = 15.3, 14.4–16.3; NICU admission (among live birth deliveries) (n = 740): yes = 26.4, 24.9–27.8; no = 14.3, 12.9–15.7.

Mean patient comorbidity score varied by level of care, with those delivering at level IV hospitals having the highest mean comorbidity scores (24.0; 95% CI = 22.4–25.6), followed by level I facilities (16.6; 95% CI = 10.9–22.3) (*p*-value < 0.001) (Figure 1). The comorbidity scores also varied in patients with several adverse outcomes. Patients with a length of stay of four or more days had higher mean comorbidity scores than those with a shorter length of stay (20.0; 95% CI = 18.9–21.0 versus 12.8; 95% CI = 10.9–14.6, *p* < 0.001). The comorbidity score increased with the number of units of blood transfused; those with 10 or more units transfused had a mean comorbidity score of 21.6 (95% CI = 18.9–24.2) compared to 17.9 (95% CI = 15.8–20.0) in those with less than four units transfused (*p* = 0.003). Finally, comorbidity score was higher among patients whose newborns were admitted to a NICU than not (26.4; 95% CI = 24.9–27.9 versus 14.3; 95% CI = 12.9–15.7, respectively; *p* < 0.001). The comorbidity index score did not vary by maternal ICU admission or transfer to a higher-level facility.

We found minimal correlation among the 27 indicators included in the Leonard Index (Table 3). The highest level of correlation was between chronic hypertension and preeclampsia with severe features (r = 0.42). All other correlation coefficients were <0.4.

We explored several additional comorbidities and risk factors among this cohort of patients with SMM that have not been previously included in obstetric risk indices but have a theoretical or demonstrated connection to SMM including fetal indicators [17], such as macrosomia, fetal growth restriction (FGR), fetal congenital anomalies, and fetal death/pregnancy loss; other maternal comorbidities, such as history of myomectomy or Loop Electrosurgical Excision Procedure (LEEP) [18] and sickle cell disease (which is grouped with anemia in the Leonard index); pregnancy-related conditions, such as nulliparity [19], hemorrhage in a prior pregnancy [17], and use of assisted reproductive technology in the index pregnancy [17]; social risk factors reported in the medical chart, such as experience of intimate partner violence, barriers to accessing health care, problems with transportation to health services, lack of stable housing, and/or language barriers [20,21]; no or late prenatal care (prenatal care initiated after the first trimester of pregnancy); and no health insurance.

In our assessment of correlations between these additional comorbidities and risk factors and the Leonard index, we found that prior LEEP or myomectomy was correlated moderately with uterine fibroids (r = 0.53), and nulliparity was negatively correlated with prior cesarean delivery (r = −0.40), both of which were expected. None of the other conditions showed high levels of correlation (Table 4).

## 4. Discussion

We applied a validated obstetric comorbidity index to a population of 978 patients with SMM identified through facility-based surveillance in Maryland. Of note, Maryland is the only state in the country with a standardized process for hospital-based SMM surveillance, benefitting from state legislation that mandates hospitals’ participation since October 2024 [22]; 26 hospitals contributed their data before this legislation came into effect, and all 32 hospitals do so currently. Like data from the various state-based maternal mortality review committees, our SMM surveillance data are considered gold-standard.

Obstetric hemorrhage was the leading cause of SMM, followed by hypertensive disorders of pregnancy, cardiovascular conditions, and infection. This study demonstrates that the validated obstetric comorbidity index proposed by Leonard et al. (2022) [13] can be applied to a cohort of patients with SMM identified through facility-based monitoring, with several minor adjustments to align conditions identified through ICD-10 codes versus electronic health record abstraction (Table A1).

Mean comorbidity scores varied significantly by primary cause of SMM, as did the pattern of common underlying conditions. Specifically, patients with SMM attributed to hypertensive disorders of pregnancy had the highest score, while those with infection had the lowest score. There are two plausible interpretations of our findings related to differences in comorbidity scores across primary cause of SMM, which may operate in tandem. These findings indicate that SMM due to certain conditions such as hemorrhage or hypertensive disorders of pregnancy are more predictable than those due to infection or cardiovascular conditions. Additionally, because infection and cardiovascular conditions are less frequent causes of SMM, the methods used to develop the Leonard index weighted more heavily conditions that predicted the more common causes of SMM (i.e., obstetric hemorrhage and hypertensive disorders or pregnancy).

Several published studies performed external validation of previously derived obstetric comorbidity indices in databases using the CDC algorithm for SMM identification. One such study sought to validate four indices, including the Leonard index, to an independent obstetrical cohort of >150,000 births [23] and found high concordance between predicted risk and actual occurrence of SMM in the Leonard index and one other index proposed about a decade earlier by Grobman et al. [9], but neither had high sensitivity for SMM at their ideal cut points. In general, because SMM is a composite outcome measure comprised of many possible complications in pregnancy or postpartum, creating a generalized comorbidity index that can adequately adjust for or predict all complications is challenging. The variation in comorbidity scores observed across primary causes of SMM helps explain why existing validated obstetric comorbidity scores tend to perform poorly at predicting the overall risk of SMM measured as a composite.

Our findings demonstrated that higher comorbidity index scores were associated with more adverse outcomes. For example, patients with a length of stay longer than four days had higher scores than those with hospital stays less than four days. Scores were also higher among patients with larger volumes of blood products transfused, as well as those where deliveries resulted in a NICU admission for the newborn. Findings showing that there were no significant differences in mean comorbidity index scores based on ICU admission or transfer to a higher level of care were unexpected. Equivalent comorbidity scores irrespective of ICU admission may reflect facility-level policies favoring ICU admission, both for clinical management in more complex SMM cases and as a precaution in less complex SMM cases. No significant difference in comorbidity scores by interhospital transfer status may represent a form of selection bias, in which patients with higher comorbidity scores are more likely to initiate care in the highest-level facilities from which patients do not transfer. In this scenario, patients being transferred from lower-level facilities may represent less predictable forms of SMM with lower comorbidity scores.

Importantly, our study identified 13 additional conditions and risk factors that may warrant further exploration for future refinements of obstetric comorbidity indices. Many of these have been noted in the prior literature to be associated with risk for SMM, including social risk factors (though not uniformly for all SMM causes) [24], hospitalization and emergency care use during pregnancy [25], the presence of fetal anomalies [26], and use of assisted reproductive technology [27]. Except for prior LEEP and myomectomy and nulliparity, none of these conditions were highly correlated with conditions already included in the Leonard index, making them good candidates for consideration in a future refined index. However, several of these conditions are not currently identifiable through existing ICD-10 codes (e.g., presence of social risk factors), diminishing the practicality of their inclusion, despite their potential importance.

### Strengths and Limitations

Our study has several limitations. Patients’ ICD-10 codes were not directly available to the research team; comorbidities and specific causes of SMM were instead identified by clinically trained abstractors from patients’ medical record, which included ICD-10 diagnosis and procedure codes. Therefore, comorbidities or diagnosis codes relevant to SMM could have been misclassified in the process of alignment with the ICD-10 codes in the Leonard index, omitted, or erroneously added to individual patient records. The high level of clinical detail in our SMM surveillance data, however, allowed us to include all patient comorbidities included in medical records through individual case reviews and data abstraction. In addition, while the Leonard index has been validated [13], the use of this index has limitations. For instance, the directionality of the relationship between select conditions included in the Leonard index, such as preterm birth, and SMM is unclear, limiting our ability to suggest causal relationships. SMM surveillance data only include information from patients who experience SMM; therefore, a comparison with the population of patients without SMM is not possible in this dataset and limits our ability to measure prevenance of risk factors in the general population. Finally, data used for this analysis are from 26 of the 32 birthing hospitals in Maryland that began contributing data voluntarily at varying time points between 2020 and 2024 before the Maryland Health Act of 2024 mandated the participation of all birthing hospitals in the state [22]. It is possible that hospitals participating voluntarily may differ systematically in ways that are associated with their SMM outcomes, although the six hospitals that did not contribute data to this analysis were smaller hospitals with few SMM events that meet our surveillance case definition. Hospitals contributing data represent >90% of births in Maryland in a given year. Though data abstraction methods are highly standardized across hospitals, we have not assessed inter-rater reliability on identifying cases of SMM or the presence of comorbidities from the EHR.

The hospital-based data source for this study is a significant strength. The level of clinical detail available, including through unstructured narrative data, far exceeds what is available in administrative or hospital discharge records and can be derived using ICD-10 codes alone. This additional detail allows for both the triangulation of data relating to patient characteristics and comorbidities and for the inclusion of variables that often have substantial missingness in administrative records, such as induction of labor and social risk factors for adverse maternal outcomes. The use of hospital-based data also allows for the operationalization of SMM using ACOG/SMFM’s definition, which has a higher positive predictive value than the criteria in the CDC algorithm for SMM identification. Further, this definition includes SMM events that occur during non-delivery antepartum hospitalizations as well as in the postpartum period, whereas the CDC algorithm is limited to delivery hospitalizations.

## 5. Conclusions

Accurately identifying patient risk for SMM has several important applications. In clinical settings, a comorbidity score can highlight the cumulative risk that is associated with a patient’s conditions. Its routine clinical use can identify patients who would benefit from close monitoring by a maternal–fetal medicine specialist or triaging high-risk patients to specialized centers that have the infrastructure, staffing, and subspecialty expertise required for a complicated pregnancy, delivery, or postpartum course. The fact that comorbidity scores vary by primary cause of SMM could assist in ensuring clinical preparedness for targeted outcomes that are more strongly associated with high comorbidity scores, such as hypertensive disorders of pregnancy or hemorrhage. Because the indices combine comorbidities into a single number, it can be used as a maternal risk-assessment tool integrated into electronic health records or used as an online calculator.

At the population or system level, comorbidity index scores can be used for case-mix adjustment necessary for comparisons of SMM rates, overall or by specific primary cause, across patient populations and hospitals. This adjustment is critical for confounding control in obstetric research and for monitoring quality improvement initiatives within and between hospitals and obstetric units.

## Figures and Tables

**Table 1:** Characteristics of the study population: Maryland Severe Maternal Morbidity Surveillance and Review Program, 2020–2024 (N = 978).

Characteristic	n	%
SMM Criteria		
ICU/CCU Admission	397	40.6
Blood Transfusion	343	35.1
Both	238	24.3
SMM Occurred During Delivery Hospitalization	774	79.1
Timing of SMM		
Antepartum	230	23.5
Intrapartum	128	13.1
Postpartum (<8 h of delivery)	448	45.8
Postpartum (8–72 h)	80	8.2
Postpartum (>72 h)	92	9.4
Maternal Age (years)		
<20	26	2.7
20–24	110	11.2
25–29	177	18.1
30–34	271	27.7
35–39	275	28.1
40+	119	12.2
Maternal Race/Ethnicity		
Non-Hispanic Asian	72	7.4
Non-Hispanic Black	434	44.4
Non-Hispanic White	308	31.5
Hispanic	133	13.6
Multi-Race/Other	17	1.7
Unknown	14	1.4
Insurance		
Public	452	46.2
Private	466	47.6
Military	17	1.7
Self-Pay/None	26	2.7
Unknown	17	1.7
Primary Cause of SMM		
Obstetric Hemorrhage	522	53.4
Hypertensive Disorders of Pregnancy	112	11.5
Infection (non-COVID)	84	8.6
Cardiovascular Condition	51	5.2
Metabolic/Endocrine Condition	32	3.3
COVID-19	30	3.1
Neurologic Condition	25	2.6
Pulmonary Condition	24	2.5
Gastrointestinal Disorder	12	1.2
Injury	10	1.0
Cancer	9	0.9
Other ^1^	32	3.3

Note: ICU/CCU = intensive care unit/critical care unit; h = hours. ^1^ Other includes anesthesia complications, adverse drug reaction, embolism, injury, renal disease, and undetermined causes.

**Table 2:** Prevalence of comorbidities and pregnancy-related conditions overall, by primary cause and timing of SMM: Maryland Severe Maternal Morbidity Surveillance and Review Program, 2020–2024 (N = 978).

	Total	Primary Cause of SMM					Timing			
		Hemorrhage	HDP	Infection	CVD	*p*-Value *	Antepartum	Intrapartum/Early Postpartum	Late Postpartum	*p*-Value **
	N = 978	N = 522	N = 112	N = 84	N = 51		N = 230	N = 576	N = 172	
**Leonard Index Risk Factors**										
PAS	77 (7.9%)	76 (14.6%)	0 (0.0%)	0 (0.0%)	0 (0.0%)	<0.001	0 (0.0%)	73 (12.7%)	4 (2.3%)	<0.001
Pulmonary hypertension	10 (1.0%)	2 (0.4%)	0 (0.0%)	1 (1.2%)	3 (5.9%)	<0.001	4 (1.7%)	5 (0.9%)	1 (0.6%)	0.440
Chronic renal disease	14 (1.4%)	7 (1.3%)	4 (3.6%)	1 (1.2%)	0 (0.0%)	0.262	3 (1.3%)	7 (1.2%)	4 (2.3%)	0.550
Cardiac disease	41 (4.2%)	13 (2.5%)	4 (3.6%)	2 (2.4%)	11 (21.6%)	<0.001	15 (6.5%)	17 (3.0%)	9 (5.2%)	0.056
HIV/AIDS	4 (0.4%)	3 (0.6%)	0 (0.0%)	0 (0.0%)	0 (0.0%)	0.700	0 (0.0%)	4 (0.7%)	0 (0.0%)	0.250
Pre-E, severe	189 (19.3%)	54 (10.3%)	87 (77.7%)	5 (6.0%)	4 (7.8%)	<0.001	43 (18.7%)	103 (17.9%)	43 (25.0%)	0.110
Placental abruption	74 (7.6%)	58 (11.1%)	14 (12.5%)	0 (0.0%)	0 (0.0%)	<0.001	15 (6.5%)	53 (9.2%)	6 (3.5%)	0.036
Bleeding disorder	27 (2.8%)	16 (3.1%)	3 (2.7%)	1 (1.2%)	1 (2.0%)	0.781	5 (2.2%)	19 (3.3%)	3 (1.7%)	0.450
Anemia	294 (30.1%)	158 (30.3%)	26 (23.2%)	31 (36.9%)	13 (25.5%)	0.182	63 (27.4%)	181 (31.4%)	50 (29.1%)	0.500
Placenta previa	56 (5.7%)	51 (9.8%)	2 (1.8%)	1 (1.2%)	0 (0.0%)	<0.001	8 (3.5%)	45 (7.8%)	3 (1.7%)	0.003
Neuromuscular	33 (3.4%)	6 (1.1%)	2 (1.8%)	1 (1.2%)	0 (0.0%)	0.808	15 (6.5%)	15 (2.6%)	3 (1.7%)	0.009
Asthma	164 (16.8%)	69 (13.2%)	23 (20.5%)	14 (16.7%)	14 (27.5%)	0.020	50 (21.7%)	78 (13.5%)	36 (20.9%)	0.005
Pre-E, non-severe/gHTN	133 (13.6%)	60 (11.5%)	34 (30.4%)	4 (4.8%)	8 (15.7%)	<0.001	22 (9.6%)	75 (13.0%)	36 (20.9%)	0.004
Connective tissue or AI	18 (1.8%)	11 (2.1%)	0 (0.0%)	0 (0.0%)	1 (2.0%)	0.243	4 (1.7%)	11 (1.9%)	3 (1.7%)	0.980
Uterine fibroids	75 (7.7%)	54 (10.3%)	8 (7.1%)	3 (3.6%)	2 (3.9%)	0.095	5 (2.2%)	58 (10.1%)	12 (7.0%)	<0.001
Substance use disorder	139 (14.2%)	56 (10.7%)	20 (17.9%)	14 (16.7%)	13 (25.5%)	<0.001	53 (23.0%)	66 (11.5%)	20 (11.6%)	<0.001
GI disorder	37 (3.8%)	16 (3.1%)	5 (4.5%)	5 (6.0%)	3 (5.9%)	0.459	9 (3.9%)	21 (3.6%)	7 (4.1%)	0.960
Chronic hypertension	175 (17.9%)	74 (14.2%)	40 (35.7%)	13 (15.5%)	13 (25.5%)	<0.001	43 (18.7%)	90 (15.6%)	42 (24.4%)	0.029
Mental health disorder	310 (31.7%)	158 (30.3%)	37 (33.0%)	24 (28.6%)	17 (33.3%)	0.878	78 (33.9%)	174 (30.2%)	58 (33.7%)	0.490
Preexisting diabetes	73 (7.5%)	22 (4.2%)	15 (13.4%)	6 (7.1%)	2 (3.9%)	<0.001	24 (10.4%)	31 (5.4%)	18 (10.5%)	0.012
Thyrotoxicosis	12 (1.2%)	6 (1.1%)	1 (0.9%)	0 (0.0%)	0 (0.0%)	0.666	2 (0.9%)	6 (1.0%)	4 (2.3%)	0.350
Gestational diabetes	109 (11.1%)	60 (11.5%)	13 (11.6%)	9 (10.7%)	4 (7.8%)	0.882	19 (8.3%)	72 (12.5%)	18 (10.5%)	0.210
BMI 40+	70 (7.2%)	27 (5.2%)	17 (15.2%)	7 (8.3%)	4 (7.8%)	<0.001	16 (7.0%)	33 (5.7%)	21 (12.2%)	0.015
Preterm birth	313 (32.0%)	145 (27.8%)	60 (53.6%)	21 (25.0%)	15 (29.4%)	<0.001	65 (28.3%)	203 (35.2%)	45 (26.2%)	0.031
Multiple pregnancy	58 (5.9%)	27 (5.2%)	11 (9.8%)	4 (4.8%)	4 (7.8%)	0.253	14 (6.1%)	36 (6.2%)	8 (4.7%)	0.730
Prior cesarean delivery	289 (29.6%)	191 (36.6%)	18 (16.1%)	20 (23.8%)	11 (21.6%)	<0.001	47 (20.4%)	197 (34.2%)	45 (26.2%)	<0.001
Maternal age 35+	394 (40.3%)	238 (45.6%)	40 (35.7%)	20 (23.8%)	16 (31.4%)	<0.001	79 (34.3%)	262 (45.5%)	53 (30.8%)	<0.001
No index comorbidities	54 (5.5%)	29 (5.6%)	0 (0.0%)	9 (10.7%)	4 (7.8%)	0.009	19 (8.3%)	28 (4.9%)	7 (4.1%)	0.110
**Mean index score (SD)**	**18.1 (15.0)**	**18.2 (16.4)**	**26.2 (11.5)**	**11.8 (9.2)**	**16.9 (15.0)**	<0.001	**15.7 (12.0)**	**19.5 (16.4)**	**16.8 (13.4)**	**0.002**
**Additional Risk Factors**										
Fetal macrosomia	39 (4.0%)	31 (5.9%)	1 (0.9%)	1 (1.2%)	1 (2.0%)	0.030	2 (0.9%)	33 (5.7%)	4 (2.3%)	0.003
FGR	42 (4.3%)	23 (4.4%)	12 (10.7%)	0 (0.0%)	1 (2.0%)	0.003	7 (3.0%)	28 (4.9%)	7 (4.1%)	0.510
Fetal congenital anomaly	28 (2.9%)	18 (3.4%)	1 (0.9%)	2 (2.4%)	1 (2.0%)	0.489	7 (3.0%)	18 (3.1%)	3 (1.7%)	0.620
IUFD/pregnancy loss	89 (9.1%)	56 (10.7%)	11 (9.8%)	11 (13.1%)	3 (5.9%)	0.607	32 (13.9%)	46 (8.0%)	11 (6.4%)	0.012
Myomectomy or LEEP	46 (4.7%)	34 (6.5%)	5 (4.5%)	1 (1.2%)	3 (5.9%)	0.241	2 (0.9%)	37 (6.4%)	7 (4.1%)	0.003
Sickle cell disease	16 (1.6%)	3 (0.6%)	1 (0.9%)	0 (0.0%)	0 (0.0%)	0.792	9 (3.9%)	5 (0.9%)	2 (1.2%)	0.008
Nulliparous	277 (28.3%)	135 (25.9%)	48 (42.9%)	25 (29.8%)	16 (31.4%)	0.005	71 (30.9%)	152 (26.4%)	54 (31.4%)	0.270
Hemorrhage in prior pregnancy	59 (6.0%)	48 (9.2%)	3 (2.7%)	1 (1.2%)	1 (2.0%)	0.003	7 (3.0%)	46 (8.0%)	6 (3.5%)	0.009
Use of ART	104 (10.6%)	70 (13.4%)	9 (8.0%)	2 (2.4%)	6 (11.8%)	0.017	11 (4.8%)	75 (13.0%)	18 (10.5%)	0.003
Social risk factors ^1^	83 (8.5%)	29 (5.6%)	9 (8.0%)	12 (14.3%)	3 (5.9%)	0.030	33 (14.3%)	36 (6.2%)	14 (8.1%)	<0.001
No or late prenatal care	226 (23.1%)	111 (21.3%)	22 (19.6%)	25 (29.8%)	17 (33.3%)	0.059	71 (30.9%)	114 (19.8%)	41 (23.8%)	<0.001
Pre-SMM hospitalization	556 (56.9%)	250 (47.9%)	69 (61.6%)	61 (72.6%)	36 (70.6%)	<0.001	142 (61.7%)	242 (42.0%)	172 (100.0%)	<0.001
No insurance	26 (2.7%)	9 (1.7%)	4 (3.6%)	5 (6.0%)	1 (2.0%)	0.097	10 (4.3%)	16 (2.8%)	0 (0.0%)	0.110

Notes: Numbers and percentages shown. Leonard index factors are ordered based on index weights and factor classification (Appendix A). SMM = severe maternal morbidity; HDP = hypertensive disorders of pregnancy; CVD = cardiovascular conditions; PAS = placental accreta spectrum; HIV/AIDS = human immunodeficiency syndrome/acquired immunodeficiency syndrome; Pre-E = preeclampsia; gHTN = gestational hypertension; AI = autoimmune disease; GI = gastrointestinal; BMI = body mass index in Kg/m^2^; SD = standard deviation; FGR = fetal growth restriction; IUFD = intrauterine fetal death; LEEP = loop electrosurgical excision procedure; ART = assisted reproductive technology; hosp. = hospitalization. * *p*-value based on chi-square analysis of difference in distribution between top 4 primary causes of SMM. ** *p*-value based on chi-square analysis of difference in distribution between timing of SMM. ^1^ Social risk factors include experience of intimate partner violence, barriers to accessing health care services/medication (including transportation), lack of stable housing, and language barriers.

**Table 3:** Tetrachoric correlation matrix of conditions included in the Leonard index: Maryland Severe Maternal Morbidity Surveillance and Review Program, 2020–2024 (N = 978).

		PAS	Pul. Htn	Chronic Renal Disease	Cardiac Disease	HIV/ AIDS	Pre-E, Severe	Placental Abruption	Bleeding Disorder	Anemia	Placenta Previa	Neuromuscular	Asthma	Pre-E, Non-Severe/gHTN	Con. Tissue or AI Disease	Uterine Fibroids	SUD	GI Disorder	cHTN	Mental Health Disorder	Preexisting Diabetes	Thyrotoxicosis	GDM	BMI 40+	Preterm Birth	Multiple Pregnancy	Prior Cesarean	Maternal Age 35+
PAS		1	0.01	0	0.01	0.04	−0.09	−0.07	0	0.07	0.24	−0.03	−0.01	−0.05	−0.04	0.14	−0.03	−0.04	−0.02	0	−0.05	0	0.01	−0.07	0.22	−0.06	0.31	0.08
Pul. htn		0.01	1	−0.01	0.33	0.15	0	0.01	−0.02	0.09	−0.03	−0.02	0.06	−0.04	0.14	0.01	0.02	0.03	0.09	0.04	−0.03	−0.01	0	−0.03	0.02	0.06	0.05	0.02
Chronic renal disease		0	−0.01	1	0.1	−0.01	0.16	0	0.03	0.01	−0.03	0.03	−0.01	0.03	−0.02	−0.03	−0.05	0.02	0.17	0.03	0.16	0.14	0.04	0	0.1	0.01	0	−0.01
Cardiac disease		0.01	0.33	0.1	1	0.07	0	−0.04	0.03	0.07	−0.03	0.02	0.03	−0.05	0.16	0	0.05	0.01	0.14	0.08	0.06	−0.02	−0.03	0.04	0.08	−0.01	0.03	0.06
HIV/AIDS		0.04	0.15	−0.01	0.07	1	−0.03	−0.02	−0.01	−0.01	0.12	−0.01	−0.03	−0.03	0.11	−0.02	−0.03	−0.01	0.01	−0.01	−0.02	−0.01	−0.02	−0.02	0.09	−0.02	0.06	0.01
Pre-E, severe		−0.09	0	0.16	0	−0.03	1	0.13	−0.02	−0.02	−0.09	0.05	0.03	−0.02	−0.03	0	0.01	0.03	0.42	0.03	0.16	0.04	0.07	0.13	0.22	0.03	−0.06	−0.01
Placental abruption		−0.07	0.01	0	−0.04	−0.02	0.13	1	−0.02	−0.06	0.03	−0.01	−0.02	−0.03	−0.01	−0.04	0.03	−0.04	−0.01	0	−0.01	−0.03	0.01	−0.03	0.02	−0.02	−0.01	−0.01
Bleeding disorder		0	−0.02	0.03	0.03	−0.01	−0.02	−0.02	1	0.03	−0.04	−0.03	−0.03	0.06	0.12	0.02	−0.03	0	−0.05	0.03	−0.02	0.04	−0.02	−0.02	−0.02	−0.04	0.01	−0.04
Anemia		0.07	0.09	0.01	0.07	−0.01	−0.02	−0.06	0.03	1	−0.01	−0.04	0.06	−0.03	0.08	0.05	0.03	−0.01	0.03	0.05	−0.02	−0.01	−0.03	0.01	0.05	0.05	0.03	−0.05
Placenta previa		0.24	−0.03	−0.03	−0.03	0.12	−0.09	0.03	−0.04	−0.01	1	−0.05	0.01	−0.06	−0.03	0.03	−0.04	−0.05	−0.06	0.02	0.03	0.01	0.04	0	0.16	−0.02	0.15	0.08
Neuro-muscular		−0.03	−0.02	0.03	0.02	−0.01	0.05	−0.01	−0.03	−0.04	−0.05	1	0.02	0.02	−0.03	−0.05	0.1	−0.01	0.03	0.1	0.03	−0.02	0.01	0.01	0.05	0	−0.06	−0.04
Asthma		−0.01	0.06	−0.01	0.03	−0.03	0.03	−0.02	−0.03	0.06	0.01	0.02	1	0.01	0.08	−0.04	0.12	0.07	0.08	0.24	0	0	−0.04	0.06	0.01	0.01	−0.02	−0.07
Pre-E, non-severe/gHTN		−0.05	−0.04	0.03	−0.05	−0.03	−0.02	−0.03	0.06	−0.03	−0.06	0.02	0.01	1	−0.03	−0.02	0.01	0.02	−0.05	0.08	0.02	0.01	−0.02	0.06	0.07	0.08	−0.1	−0.03
Con. tissue or AI disease		−0.04	0.14	−0.02	0.16	0.11	−0.03	−0.01	0.12	0.08	−0.03	−0.03	0.08	−0.03	1	−0.01	−0.06	0.17	0.04	0.02	−0.04	0.05	−0.05	−0.04	0	−0.03	−0.02	0
Uterine fibroids		0.14	0.01	−0.03	0	−0.02	0	−0.04	0.02	0.05	0.03	−0.05	−0.04	−0.02	−0.01	1	−0.08	0.02	0.09	−0.05	−0.04	0	0.06	−0.01	0	0.01	0.03	0.19
SUD		−0.03	0.02	−0.05	0.05	−0.03	0.01	0.03	−0.03	0.03	−0.04	0.1	0.12	0.01	−0.06	−0.08	1	0.01	0.02	0.18	−0.05	−0.02	−0.07	−0.03	0.04	0	−0.02	−0.13
GI disorder		−0.04	0.03	0.02	0.01	−0.01	0.03	−0.04	0	−0.01	−0.05	−0.01	0.07	0.02	0.17	0.02	0.01	1	−0.04	0.03	−0.04	0.03	0.01	−0.03	0.06	0.06	−0.03	0.01
cHTN		−0.02	0.09	0.17	0.14	0.01	0.42	−0.01	−0.05	0.03	−0.06	0.03	0.08	−0.05	0.04	0.09	0.02	−0.04	1	0.09	0.17	0.02	0.12	0.18	0.14	0.06	0.09	0.12
MH disorder		0	0.04	0.03	0.08	−0.01	0.03	0	0.03	0.05	0.02	0.1	0.24	0.08	0.02	−0.05	0.18	0.03	0.09	1	0.02	0	0.05	0.07	0.06	0.02	−0.03	−0.1
Preexisting diabetes		−0.05	−0.03	0.16	0.06	−0.02	0.16	−0.01	−0.02	−0.02	0.03	0.03	0	0.02	−0.04	−0.04	−0.05	−0.04	0.17	0.02	1	0	0.04	0.13	0.11	−0.01	0.05	0.03
Thyrotoxicosis		0	−0.01	0.14	−0.02	−0.01	0.04	−0.03	0.04	−0.01	0.01	−0.02	0	0.01	0.05	0	−0.02	0.03	0.02	0	0	1	−0.01	0.01	0.02	−0.03	0.01	0
GDM		0.01	0	0.04	−0.03	−0.02	0.07	0.01	−0.02	−0.03	0.04	0.01	−0.04	−0.02	−0.05	0.06	−0.07	0.01	0.12	0.05	0.04	−0.01	1	0.15	0.03	−0.01	0.02	0.14
BMI 40+		−0.07	−0.03	0	0.04	−0.02	0.13	−0.03	−0.02	0.01	0	0.01	0.06	0.06	−0.04	−0.01	−0.03	−0.03	0.18	0.07	0.13	0.01	0.15	1	0.06	0.06	0.05	0.01
Preterm birth		0.22	0.02	0.1	0.08	0.09	0.22	0.02	−0.02	0.05	0.16	0.05	0.01	0.07	0	0	0.04	0.06	0.14	0.06	0.11	0.02	0.03	0.06	1	0.16	0.18	0.04
Multiple pregnancy		−0.06	0.06	0.01	−0.01	−0.02	0.03	−0.02	−0.04	0.05	−0.02	0	0.01	0.08	−0.03	0.01	0	0.06	0.06	0.02	−0.01	−0.03	−0.01	0.06	0.16	1	−0.03	−0.01
Prior cesarean		0.31	0.05	0	0.03	0.06	−0.06	−0.01	0.01	0.03	0.15	−0.06	−0.02	−0.1	−0.02	0.03	−0.02	−0.03	0.09	−0.03	0.05	0.01	0.02	0.05	0.18	−0.03	1	0.08
Maternal age 35+		0.08	0.02	−0.01	0.06	0.01	−0.01	−0.01	−0.04	−0.05	0.08	−0.04	−0.07	−0.03	0	0.19	−0.13	0.01	0.12	−0.1	0.03	0	0.14	0.01	0.04	−0.01	0.08	1
Correlation coefficient (r)																												
							
−0.8	−0.4	−0.2	0.0	0.2	0.4	0.8																						

Note: PAS = placental accreta spectrum; Pul.htn = pulmonary hypertension; HIV/AIDS = human immunodeficiency syndrome/acquired immunodeficiency syndrome; Pre-E = preeclampsia; gHTN = gestational hypertension; Con. = connective; AI = autoimmune; SUD = substance use disorder; GI = gastrointestinal; cHTN = chronic hypertension; MH = mental health; GDM = gestational diabetes mellitus; BMI = body mass index.

**Table 4:** Tetrachoric correlation matrix of additional risk factors with Leonard index conditions: Maryland Severe Maternal Morbidity Surveillance and Review Program, 2020–2024 (N = 978).

				Macrosomia	FGR	Fetal Congenital Anomaly	Pregnancy Loss/FD	LEEP or Myomectomy	Sickle Cell Disease	Nulliparous	Prior Hemorrhage	Use of ART	Social Risk Factors	Late or No Prenatal Care	Pre-SMM Hops.	No Health Insurance
PAS				−0.06	0.02	0.05	−0.04	0.19	−0.04	−0.11	0.12	0.14	−0.04	−0.05	0.00	0.00
Pulmonary hypertension				−0.02	−0.02	−0.02	0.01	0.03	0.24	0.01	−0.03	0.00	−0.03	0.00	0.06	−0.02
Chronic renal disease				−0.03	0.07	0.03	−0.04	−0.03	−0.02	0.03	−0.03	−0.01	−0.04	−0.04	0.06	0.04
Cardiac disease				−0.04	0.01	0.03	−0.01	0.00	0.05	−0.04	−0.01	−0.02	−0.01	0.01	0.09	−0.03
HIV/AIDS				−0.01	−0.01	−0.01	−0.02	−0.01	−0.01	−0.04	−0.02	0.03	−0.02	0.00	−0.01	−0.01
Preeclampsia, severe				−0.03	0.12	−0.02	0.03	−0.04	−0.02	0.10	0.00	−0.06	0.02	−0.02	0.08	−0.01
Placental abruption				−0.04	0.05	0.00	0.36	0.04	−0.04	−0.02	−0.02	−0.08	0.01	−0.02	−0.04	0.03
Bleeding disorder				0.00	0.00	−0.03	0.00	0.03	−0.02	0.00	0.07	0.01	−0.05	−0.01	−0.01	−0.03
Anemia				−0.02	0.00	0.01	−0.02	0.05	0.20	−0.02	0.06	−0.08	0.04	0.02	0.07	−0.02
Placenta previa				−0.03	−0.03	0.01	−0.06	0.01	−0.03	−0.12	0.09	0.06	−0.03	−0.04	0.04	−0.01
Neuromuscular				−0.01	0.02	0.00	−0.01	−0.01	−0.03	−0.03	0.00	−0.01	0.14	0.00	0.06	−0.03
Asthma				−0.03	−0.02	0.01	0.01	0.00	0.07	0.01	0.01	−0.08	0.07	0.03	0.14	−0.06
Preeclampsia, non-severe/gHTN				0.00	0.01	−0.01	−0.08	0.00	0.02	0.12	−0.06	0.06	0.01	0.03	0.10	−0.01
Connective tissue or AI disease				0.01	−0.03	−0.02	−0.01	−0.03	−0.02	−0.02	0.06	−0.02	−0.04	−0.02	0.02	−0.02
Uterine fibroids				0.00	−0.04	0.00	−0.02	0.53	−0.04	0.11	0.10	0.17	−0.07	−0.08	−0.05	−0.02
Substance use disorder				−0.08	0.04	−0.05	0.10	−0.06	0.07	−0.07	−0.01	−0.14	0.24	0.21	0.05	−0.05
GI disorder				0.01	−0.02	0.00	−0.02	−0.02	−0.03	0.00	−0.05	0.04	0.00	−0.03	0.02	0.00
Chronic hypertension				−0.03	0.04	0.00	−0.02	0.06	−0.06	−0.03	0.08	−0.05	0.01	0.01	0.12	−0.01
Mental health disorder				0.02	−0.03	−0.02	−0.02	−0.06	0.02	0.04	−0.04	−0.05	0.07	0.00	0.10	−0.10
Preexisting diabetes				0.03	0.05	−0.02	−0.04	−0.02	0.00	−0.02	−0.02	−0.04	0.07	0.01	0.10	0.01
Thyrotoxicosis				−0.02	−0.02	−0.02	0.00	−0.03	−0.02	−0.03	0.01	−0.01	0.00	−0.04	0.06	0.04
Gestational diabetes				0.05	−0.01	−0.02	−0.06	0.04	−0.02	0.01	−0.02	0.02	−0.03	−0.07	0.01	0.07
BMI 40+				0.00	−0.04	−0.02	−0.05	−0.02	−0.04	0.00	0.00	−0.02	−0.02	0.01	0.06	0.01
Preterm birth				−0.10	0.09	0.01	−0.20	0.03	−0.02	−0.08	0.04	0.00	0.03	−0.02	0.13	−0.01
Multiple pregnancy				−0.05	0.08	0.04	0.02	0.05	0.04	0.05	0.01	0.06	0.01	−0.03	0.03	−0.04
Prior cesarean delivery				0.05	−0.03	0.04	0.00	0.03	−0.03	−0.40	0.14	−0.04	−0.01	0.01	0.00	0.01
Maternal age 35+				0.04	−0.02	0.08	−0.01	0.17	−0.04	−0.11	−0.01	0.27	−0.11	−0.05	−0.09	−0.05
Macrosomia				1.00	−0.04	−0.04	−0.06	−0.02	−0.03	0.01	0.04	0.05	−0.02	−0.05	−0.08	0.03
FGR				−0.04	1.00	0.06	0.03	−0.02	−0.03	0.01	0.01	−0.02	0.01	−0.03	−0.05	0.00
Fetal congenital anomaly				−0.04	0.06	1.00	0.04	0.05	−0.02	−0.04	−0.02	0.04	−0.05	−0.03	−0.01	−0.03
Pregnancy loss/IUFD				−0.06	0.03	0.04	1.00	0.03	−0.04	−0.03	−0.01	−0.05	0.08	0.05	−0.02	0.02
LEEP or myomectomy				−0.02	−0.02	0.05	0.03	1.00	−0.03	0.03	0.07	0.26	−0.07	−0.09	−0.02	−0.04
Sickle cell disease				−0.03	−0.03	−0.02	−0.04	−0.03	1.00	0.04	−0.03	−0.05	−0.01	0.01	0.11	−0.02
Nulliparous				0.01	0.01	−0.04	−0.03	0.03	0.04	1.00	−0.15	0.10	0.02	−0.07	−0.04	−0.04
Prior hemorrhage				0.04	0.01	−0.02	−0.01	0.07	−0.03	−0.15	1.00	0.00	−0.03	0.01	−0.04	−0.01
Use of ART				0.05	−0.02	0.04	−0.05	0.26	−0.05	0.10	0.00	1.00	−0.11	−0.14	0.01	−0.06
Social risk factors				−0.02	0.01	−0.05	0.08	−0.07	−0.01	0.02	−0.03	−0.11	1.00	0.15	0.06	0.05
Late or no prenatal care				−0.05	−0.03	−0.03	0.05	−0.09	0.01	−0.07	0.01	−0.14	0.15	1.00	−0.05	0.07
Pre-SMM hospitalization				−0.08	−0.05	−0.01	−0.02	−0.02	0.11	−0.04	−0.04	0.01	0.06	−0.05	1.00	0.04
No health insurance				0.03	0.00	−0.03	0.02	−0.04	−0.02	−0.04	−0.01	−0.06	0.05	0.07	0.04	1.00
Correlation coefficient (r)																
							
−0.8	−0.4	−0.2	0.0	0.2	0.4	0.8										

Note: PAS = placental accreta spectrum; HIV/AIDS = human immunodeficiency syndrome/acquired immunodeficiency syndrome; AI = autoimmune; GI = gastrointestinal; BMI = body mass index; FGR = fetal growth restriction; IUFD = intrauterine fetal death; LEEP = loop electrosurgical excision procedure; ART = assisted reproductive technology; SMM = severe maternal morbidity.

## Data Availability

The datasets presented in this article are not readily available because they include confidential and protected information.

## Abbreviations

The following abbreviations are used in this manuscript:

ACOG	American College of Obstetricians and Gynecologists
CDC	Centers for Disease Control and Prevention
CCU	Critical Care Unit
ICD	International Classification of Diseases
ICU	Intensive Care Unit
LEEP	Loop Electrosurgical Excision Procedure
NICU	Neonatal Intensive Care Unit
SMFM	Society for Maternal and Fetal Medicine
SMM	Severe Maternal Morbidity

## Appendix A

**Table A1:** Classification of comorbidity index conditions in the Severe Maternal Morbidity Surveillance System.

Comorbidity (Index Weight)	Classification	ICD-10-CM (Leonard Index)	SMM Surveillance Conditions
Placenta accreta spectrum (27)	Comorbidity	O43.213, O43.223, O43.233	Placenta accreta spectrum
Pulmonary hypertension (20)	Comorbidity	I27.0, I27.2	Pulmonary hypertension
Chronic renal disease (17)	Comorbidity	O26.83, I12, I13, N03-N05, N07, N08, N11.1, N11.8, N11.9, N18, N25.0, N25.1, N25.81, N25.89, N25.9, N26.9	Chronic renal disease
Cardiac disease, preexisting (14)	Comorbidity	I05-I09, I11-I13, I15, I16, I20, I25, I27.8, I30-I41, I44-I49, I50.22, I50.23, I50.32, I50.33, I50.42, I50.43, I50.812, I50.813 O99.41, O99.42, Q20-Q24	Congenital and preexisting acquired cardiac disease including cardiac valvular disease, chronic congestive heart failure, chronic ischemic heart disease, myocardial infarction
HIV or AIDS (13)	Comorbidity	O98.7, B20	HIV or AIDS
Preeclampsia with severe features (12)	Comorbidity	O14.1, O14.2, O11	Preeclampsia with severe features, superimposed preeclampsia
Placental abruption (9)	Comorbidity	O45	Placental abruption
Bleeding disorder, preexisting (9)	Comorbidity	D66, D67, D68.0-D68.6, D69	Bleeding disorders including antiphospholipid syndrome, factor V Leiden, factor 11 deficiency, hemophilia, thrombocytopenia, Von Willebrand disease
Anemia, preexisting (9)	Comorbidity	O99.01, O99.02, D50, D55, D56, D58, D59, D57.1, D57.20, D57.3, D57.40, D57.80	Preexisting anemia, thalassemia, sickle cell disease, sickle cell trait
Placenta previa, complete or partial (8)	Comorbidity	O44.03, O44.13, O44.23, O44.33	Placenta previa
Neuromuscular disease (6)	Comorbidity	G40, G70	Neuromuscular disease including epilepsy and myasthenia gravis
Asthma, acute or moderate-severe (5)	Comorbidity	O99.5, J45.21, J45.22, J45.31, J45.32, J45.4, J45.5, J45.901, J45.902	Asthma
Preeclampsia without severe features or gestational hypertension (5)	Comorbidity	O13, O14.0, O14.9	Preeclampsia without severe features, gestational hypertension, unspecified preeclampsia
Connective tissue or autoimmune disease (4)	Comorbidity	M30-M36	Connective tissue disorders including anti-synthetase syndrome, Ehlers–Danlos syndrome, Sjogren’s syndrome,
Uterine fibroids (4)	Comorbidity	D25, O34.1	Uterine fibroids
Substance use disorder (4)	Comorbidity	F10-F19, O99.31, O99.32	Substance use disorder including use of the following substances during index pregnancy: alcohol, tobacco, cannabis, opioids, methamphetamines, prescription drugs (not as intended), opioid agonist therapy), other illicit substances
Gastrointestinal disease (3)	Comorbidity	K50-K52, K70-K77, K80-K83, K85-K87, K94, K95, O26.6	Gastrointestinal diseases including cholestasis, fatty liver disease
Chronic hypertension (3)	Comorbidity	O10, O11, I10	Chronic hypertension
Major mental health disorder (3)	Comorbidity	F06, F20-F25, F28-F34, F39, F40.0, F41, F43, F53, F60	Major mental health disorders including adjustment disorder, anxiety, borderline personality disorder, depression, mood disorders, postpartum depression, post-traumatic stress disorder, schizophrenia
Preexisting diabetes mellitus (2)	Comorbidity	E08-E13, O24.0, O24.1, O24.3, O24.8, O24.9, Z79.4	Type 1 diabetes, Type 2 diabetes, unspecified diabetes
Thyrotoxicosis (2)	Comorbidity	E05	Thyrotoxicosis, hyperthyroid disorders
Gestational diabetes mellitus (1)	Comorbidity	O24.4	Gestational diabetes mellitus
BMI (kg/m^2^) at delivery 40 or greater (1)	Comorbidity	Birth certificate—use Z68.4, E66.01, E66.2 if birth certificate not available	Pre-pregnancy BMI 40 or greater
Preterm birth (less than 37 weeks) (8)	Pregnancy-related condition	Z3A.20-Z3A.36	Birth at gestational age < 37 weeks
Twin or multiple pregnancy (9)	Pregnancy-related condition	O30, O31, Z37.2-Z37.7	Twin or higher order pregnancy
Previous cesarean birth (2)	Pregnancy-related condition	O34.21	Previous cesarean birth
Maternal age 35 y or older (0)	Pregnancy-related condition	Birth certificate/Discharge record	Maternal age 35 years or older at admission
